# E40, a novel microbial protease efficiently detoxifying gluten proteins, for the dietary management of gluten intolerance

**DOI:** 10.1038/s41598-019-48299-7

**Published:** 2019-09-11

**Authors:** Linda Cavaletti, Anna Taravella, Lucia Carrano, Giacomo Carenzi, Alessandro Sigurtà, Nicola Solinas, Salvatore De Caro, Luigia Di Stasio, Stefania Picascia, Mariavittoria Laezza, Riccardo Troncone, Carmen Gianfrani, Gianfranco Mamone

**Affiliations:** 1 Fondazione Istituto Insubrico di Ricerca per la Vita (FIIRV), Gerenzano, Varese Italy; 2 Institute of Food Sciences, National Research Council, Avellino, Italy; 3 Institute of Biochemistry and Cell Biology, National Research Council, Naples, Italy; 4 Department of Translational Medical Science (Section of Pediatrics), and European Laboratory for the Investigation of Food-Induced Diseases, University “Federico II”, Naples, Italy

**Keywords:** Coeliac disease, Microbiology, Proteases

## Abstract

Gluten proteins are the causative agent of Celiac Disease (CD), a life-long food intolerance characterized by an autoimmune enteropathy. Inadvertent gluten exposure is frequent even in celiac patients complying with a gluten-free diet, and the supplementation of exogenous gluten-digestive enzymes (glutenases) is indeed a promising approach to reduce the risk of dietary gluten boost. Here we describe Endopeptidase 40, a novel glutenase discovered as secreted protein from the soil actinomycete *Actinoallomurus* A8, and its recombinant active form produced by *Streptomyces lividans* TK24. E40 is resistant to pepsin and trypsin, and active in the acidic pH range 3 to 6. E40 efficiently degrades the most immunogenic 33-mer as well as the whole gliadin proteins, as demonstrated by SDS-PAGE, HPLC, LC-MS/MS, and ELISA. T lymphocytes from duodenal biopsies of celiac patients showed a strongly reduced or absent release of IFN-γ when exposed to gluten digested with E40. Data in gastrointestinal simulated conditions suggest that no toxic peptides are freed during gluten digestion by E40 into the stomach to enter the small intestine, thus counteracting the intestinal inflammatory cascade to occur in CD patients. E40 is proposed as a novel candidate in Oral Enzymatic Therapy for the dietary management of gluten toxicity.

## Introduction

Celiac Disease (CD) is a chronic autoimmune enteropathy, with a defined environmental trigger in the form of gluten proteins contained in wheat and related cereals^1^. Gluten intolerance affects genetically predisposed patients carrying the HLA DQ2/DQ8 genes, with a prevalence estimated approximately 1% in wheat consuming countries^1^. The small intestinal mucosa of subjects with acute CD shows increased numbers of intraepithelial lymphocytes, villous atrophy, crypts hyperplasia, and a chronic inflammatory status^2^. Gluten is a heterogeneous mixture of insoluble proteins, consisting of gliadins and glutenins present in wheat, rye and barley cereals^3^. The high proline and glutamine residue content renders the gluten proteins largely inaccessible to human proteases of the gastrointestinal tract. This allows large proline/glutamine rich peptides to reach the small intestine and trigger immune responses in patients with CD^4,5^. These undigested peptides can cross the small intestine mucosal barrier reaching the lamina propria where they are deamidated by tissue transglutaminase (tTG)^6^. The deamidated gluten peptides do especially trigger both humoral and T-cell mediated adaptive immune responses^7^. To date, several T-cell stimulatory peptides, resistant to gastrointestinal digestion, have been identified either in gliadin and glutenin proteins^8^, though a 33-mer from α-gliadin is currently considered the most immunogenic peptide, as it includes six overlapping epitopes^4^.

Life-long adherence to a strict gluten-free diet (GFD) is the only effective treatment available for CD patients^9^. However, many patients fail to respond to GFD either clinically or histologically. Several surveys highlight the psychosocial implications of adherence to a GFD, hence, efforts are ongoing to develop supplemental strategies to support GFD or therapeutic approaches beyond the GFD^10^. Total gluten avoidance is pragmatically impossible due to gluten ubiquity in food industry, even in CD subjects strictly committed to the GFD^10,11^. Novel therapies, aimed at rendering gluten harmless and improving CD patient quality of life, have been intensively investigated^12^. A major area of research is the development of enzyme preparations able to digest the toxic fragments of gluten in the stomach, thus preventing exposure of these fragments to the intestinal mucosa. To this end, endopeptidases produced by various plants, bacteria, or fungi have been studied and demonstrated to degrade at different extent the proline/glutamine rich gluten peptides in the gastric and upper intestinal tracts, thus “detoxifying” gluten^13^. These novel enzymes, termed “*glutenases*”, could be devised as oral supplements, supporting the GFD and protecting from unintentional gluten exposures. Previous *in vitro* studies demonstrated that some of these glutenases are efficient in catalyzing the digestion of gluten proteins in acidic condition, reporting degradation of up to 70–99% of gluten protein load^13,14^. All the experimental conditions set-up to assess gluten detoxification forecast to test a glutenase in combination with the gastric enzyme pepsin, to obtain a complete gluten degradation in the first gastrointestinal tract^14^. As the main goal of the Oral Enzymatic Therapy (OET) is to rapidly detoxify gluten proteins and, above all, preferably during the transit in the stomach, the availability of proteases directly and efficiently detoxifying gluten even irrespective of pepsin partial gluten-digestion, would be very promising to this respect^15^.

Here, we present the acid and pepsin-resistant Endoprotease 40 (E40), a novel glutenase originally discovered as secreted protease from the acidophilic actinomycete *Actinoallomurus* A8 (International patent WO 2013/083338). Actinomycetes, a group of soil inhabitant filamentous bacteria, are an interesting source of proteases^16,17^. In particular, acidophilic actinomycete strains, which grow in acidic environments, are expected to express extracellular proteases with enzymatic properties suitable for OET in CD^18–20^. E40 was expressed as recombinant protein in *Streptomyces lividans* TK24 strain and its glutenase activity was comprehensively assessed by proteomic, enzymatic and immunological approaches. The data gathered are critically discussed and statistically evaluated with the final aim to demonstrate the potentiality of E40 as a novel candidate for OET in CD.

## Results

### Discovery of endopeptidase E40 from the actinomycete *Actinoallomurus* A8 and assessment of its glutenase activity

**Figure 1:**
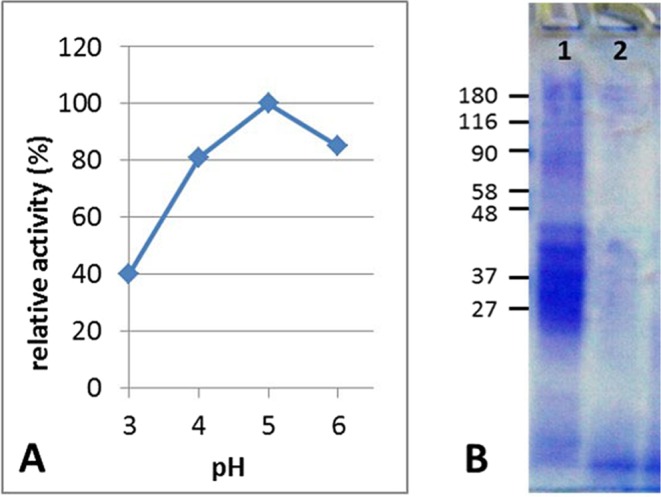
Activity of *Actinoallomurus* A8 culture supernatant fraction “<100 kDa” containing the wild Endopep-40. (**A**) Activity toward the substrate sucAlaAlaProPhe-AMC at various pH values. X axis: pH value, Y axis: relative activity (%). (**B**) Gliadin digestion by the wild E40 containing supernatant fraction after 2 hour of incubation at 37 °C, pH 3. Gel stained with Coomassie R250 brilliant blue. Lane 1: gliadin alone, lane 2: gliadin + E40 (<100 kDa fraction).

**Figure 2:**
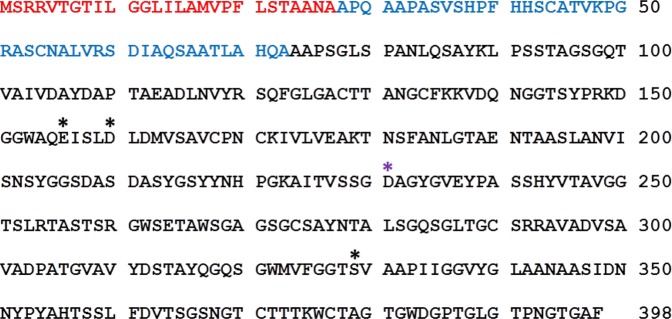
Aminoacid sequence of E40. Sequence labeled with aminoacid numbering from the N-terminus of the whole protein. 1–27 (red): putative signal peptide sequence (according to signalP 4.1 server prediction), 28–73 (blue): putative pro-enzyme sequence, 74–398 (black): mature form. Aminoacids partecipating to the active site are indicated by asterisks; in black, the putative catalytic triad.

E40 was discovered as a new secreted protease from a newly isolated strain, labeled A8, assessed by 16S rRNA gene sequencing to belong to the genus *Actinoallomurus*, whose members grow optimally at pH values close to 5^21^. *Actinoallomurus* A8 was isolated from a soil with pH 4.2 collected in the north of Italy (supplementary material, Figs S1 and S2). E40 purified from A8 culture supernatant (corresponding to “<100 kDa” fraction), had an enzymatic activity in the range of pH 3–6 with optimum at pH 5 (Fig. 1, panel A). At this environmental condition, E40 showed a marked glutenase activity since gliadin proteins were completely digested (Fig. 1, panel B). E40 coding gene identification (e40, accession number MK303398, Fig. S3) was carried out by genome sequence of whole *Actinoallomururs* A8 genome and by MudPit analysis of the “<100 kDa” fraction. The resulting E40 whole gene translation predicted a protein consisting of 398 amino acid residues, belonging to the serine-carboxyl peptidase S53 family (as revealed by Blast analysis), with the catalytic triad formed by aspartic acid, glutamic acid and serine at positions 156, 160 and 329, respectively (Fig. 2). Notably, the presence of glutamic acid instead of histidine, which is typical of the serine proteases catalytic triad, and the presence of a further aspartic residue in the active site at position 231, confers E40 the capability to hydrolyze at low pH value^22^. The *signalP 4*.*1* server analysis identified the N-terminal signal peptide between positions 27 and 28, and predicted that the mature form started from position 74, resulting in an expected 32.5 kDa mature enzyme (Fig. 2).

### Cloning and expression of E40 coding gene in *S*. *lividans* TK24/plJ86

**Figure 3:**
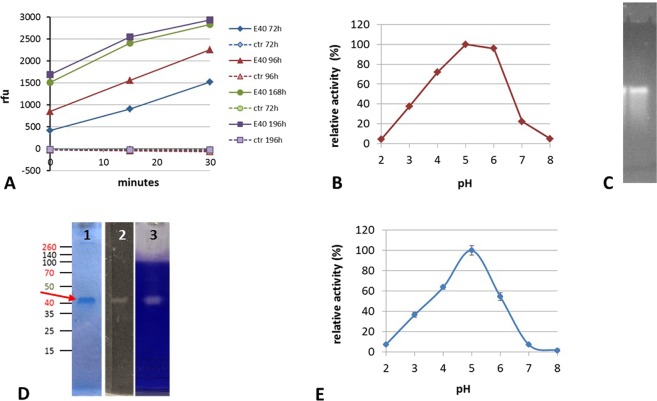
Expression of E40 as active secreted protease by *S*. *lividans* TK24/plJ86/e40 submerged fermentation at flask scale (panels (A–C)) or in 15 L bioreactor, with subsequent E40 purification (panels (D and E)). (**A**) Activity of supernatant samples taken at different fermentation times from the producing *S*. *lividans* TK24/plJ86/E40 (E40, continuous line) and absence of activity from the plJ86-empty control strain (ctr, dashed lines). ◆72, ▴96, •168, ◾192 fermentation hours. Activity toward the substrate sucAAPF-AMC at pH5 is shown as relative fluorescent units (Y axis) produced in time (X axis). (**B**) Activity showed by the 168 hours supernatant (SN) sample at different pH, showing the optimum at pH5. (**C**) Zymograhy of the 168 h sample at pH5, using the same substrate as in panel (A) and visualized under UV light. (**D**) Profile of purified E40 from the 15 L fermentation, lane 1: final E40 preparation stained with Coomassie (the red arrow indicates E40), lane 2: zymography as in (**C**), lane 3: band of gelatin hydrolysis after 2 hours of digestion at 37 °C and pH5, visualized after Coomassie staining of the gelatin substrate.

E40 coding gene was successfully cloned in *Streptomyces lividans* TK24, using the pIJ86 expression vector (Fig. S4). The recombinant E40 (rE40) producing *S*. *lividans* TK24/pIJ86/e40 was cultivated in an animal origin supplement-free medium. The monitoring of the enzymatic activity in culture supernatant at flask level demonstrated a stable and continuous production of rE40, up to 8 days of fermentation (Fig. 3, panel A). As per its wild form, rE40 was active in the pH range 3–6, with an optimum at pH 5 (Fig. 3, panel B). The zymographic analysis, carried out at pH 5, demonstrated that rE40 was the only active protein in culture supernatants (Fig. 3, panel C).

The final powder obtained after the fermentation and the downstream purification process scaled at 15 L volume, contained 6% total proteins with specific rE40 activity of 41.000 U/mg. The purified rE40 had chemical/physical properties and bioactivity as per the wild-type form. The efficiency of the purification process was demonstrated by SDS-PAGE, where rE40 migrated in the main band at ~40 kDa (Fig. 3, panel D, lane 1, red arrow) and was the only protein active toward the chromogenic substrate suc-Ala-Ala-Pro-Phe-AMC (Fig. 3, panel D, lane 2). The N-terminal sequence analysis of the 40 kDa SDS-PAGE band confirmed the predicted mature form of rE40. rE40 was also the only protein active against a generic protein substrate like gelatin (Fig. 3, panel D, lane 3), thus excluding the presence of any endoprotease other than rE40 in the final enzymatic preparation. Activity of purified rE40 at different pH values was evaluated with the substrate suc-Ala-Ala-Pro-Phe-pNA, pH range 2 to 8 (Fig. 3, panel E). It resulted to be maximally active at pH 5 and significant residual activity (~40%) was maintained at pH 3. These results predict optimal action in a post-prandial gastric environment, when pH keeps higher than 3 for over an hour^23,24^. Interestingly, rE40 remains significantly active (approximately 50%) at pH 6, suggesting also a prolonged action into the post-prandial duodenal compartment where pH reduces to pH ≤ 6 for long time, after the meal^24^. Overall, our results support a perfect similarity between the wild and recombinant form of E40. For such reason and easiness of reading, the glutenase will be referred to as E40 in the following text.

### E40 activity is resistant at *in vitro* gastrointestinal digestive milieu

**Figure 4:**
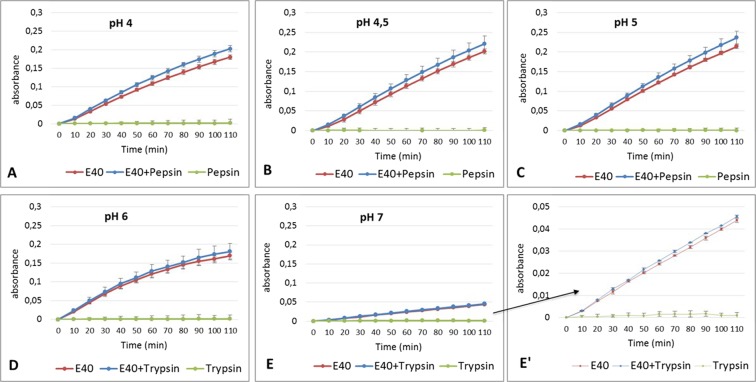
Activity of E40 evaluated in absence or in presence of digestive proteases pepsin (P) or trypsin (T) at pH 4, 4.5 and 5 for P (panels (A–C)) or pH 6 and 7 for T (panels (D and E)), respectively. Enzymes and substrate solutions were separately incubated at 37 °C for 5 minutes, the reactions were started by addition of the substrate to enzyme samples of E40 alone (red line), E40 with P or T (blue line), and P or T alone (green line). Each reaction condition was tested in duplicate; error bars represent the standard deviation. Final concentrations in reaction mix were: 200 µM substrate, 1–4 nM E40 for incubations at pH 4 to 5 or 4 nM and 7 nM for incubations at pH 6 or 7, respectively (E40 concentration was adjusted according to optimum pH). The digestive protease were maintained in the ratio was 1:2 (w/w) versus E40. Panel (E’) is a magnification of E. X axis: incubation time (minutes), Y axis: absorbance units read at 410 nm. (R^2^ values are 0.9948, 0.9976, 0.9978, 0.9639, 0.9994 for E40 alone and 0.993, 0.9975, 0.9982: 0.9661, 0.9994 for E40 + P/T for pH 4, 4.5, 5, 6 and 7, respectively).

The resistance of E40 to the proteolytic activity of gastric (pepsin) and duodenal (trypsin) digestive proteases was further evaluated (Fig. 4), using suc-Ala-Ala-Pro-Phe-pNA as substrate. Chymotrypsin was not included in the analysis, because active onto the same substrate. Noteworthy, either pepsin (Fig. 4, panels A–C) or trypsin (Fig. 4, panels D and E) did not hydrolyze the chromogenic substrate. E40 activity was assessed with and without pepsin (1:2 w/w ratio *vs* E40) in the pH range 4–5, and with or without trypsin (1:2 w/w ratio *vs* E40) in pH 6–7. Free pNA release from hydrolysis of the chromogenic substrate was monitored during reactions protracted for 110 minutes at 37 °C. Notably, E40 cleaving activity was unaffected either by pepsin/trypsin addition or by acidic pH. By contrast, a slightly enhanced E40 activity was observed in the presence of pepsin (Fig. 4, panels A–C), maybe due to unmasking of E40 catalitic site by pepsin. Overall, these findings confirm E40 resistance to the main human digestive proteases, at physiological conditions.

### Pronounced digestion of the immunodominant 33-mer peptides and gliadin proteins by E40

**Figure 5:**
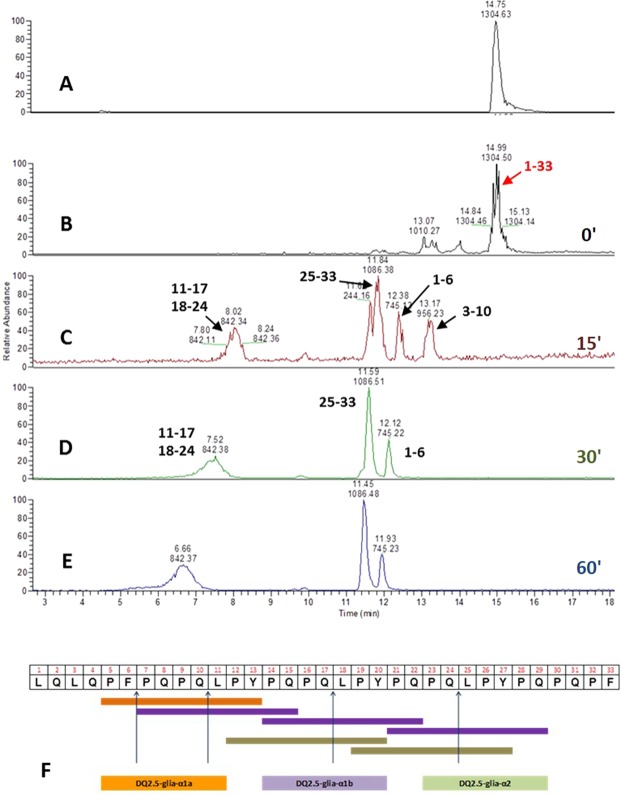
E40 completely removes the immunogenicity of the 33-mer peptide. LC/MS profile of 33-mer digestion by E40 at pH 5 and 37 °C, with E40 in a molar ratio 1:48 *versus* peptide. Panel (A): Trace of full length peptide alone (triply charged ion [(M + 3 H^+^)^3+^ = 1304.68]). Panels (B–E): Time-course of 33-mer cleavage by E40 activity (from to top-bottom 0, 15, 30, 60 minutes digestion, respectively). Peptide fragments formed during digestion are indicated by arrows. Rapid action of E40 onto the substrate can be visible at time 0′, complete disappearance of 33-mer is obtained already after 15′ of digestion. Panel (F): Schematic visualization of E40 cleavage sites deduced from the final residual peptides which demonstrates the complete epitopes destruction since 30′ reaction.

**Figure 6:**
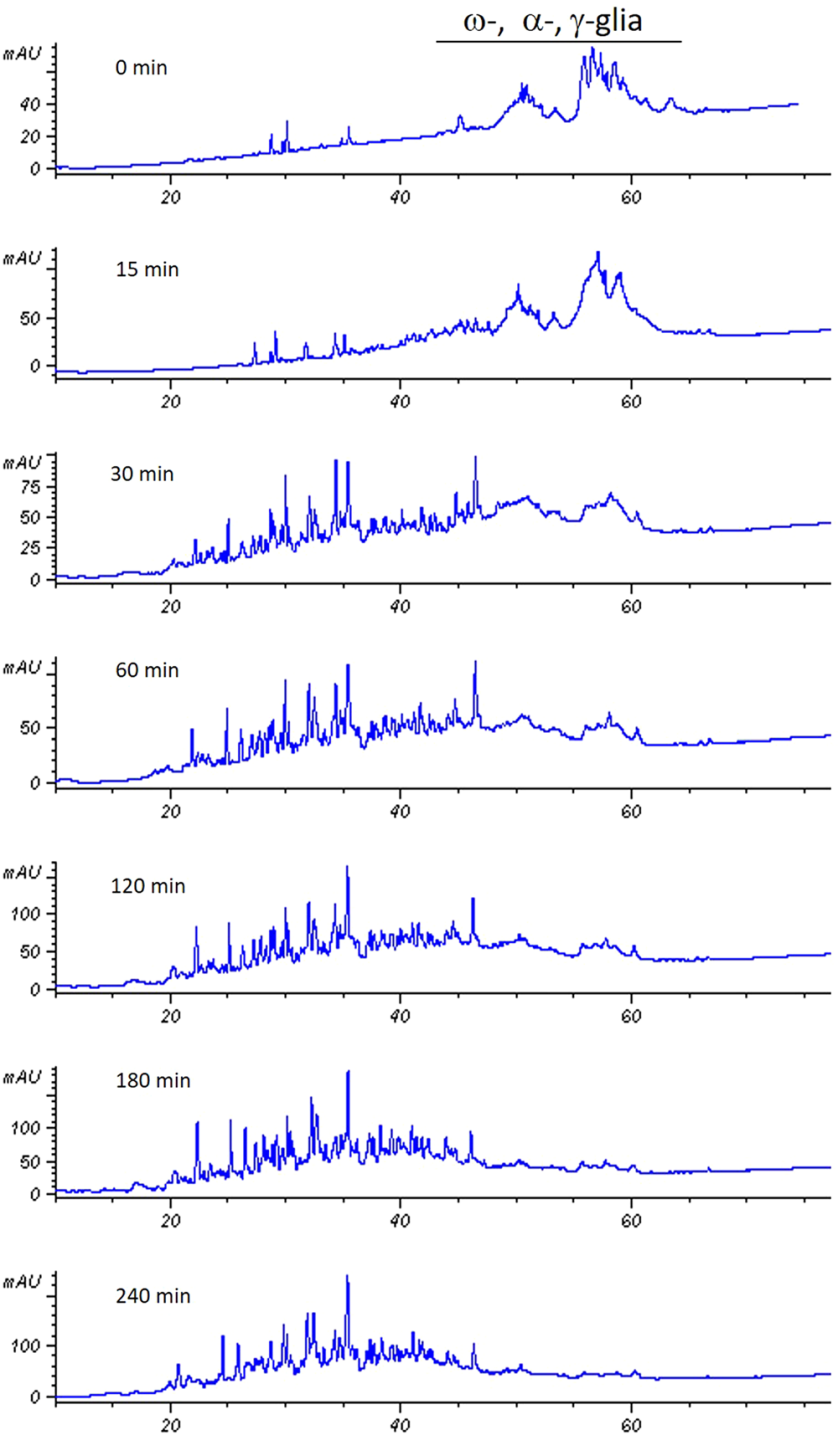
HPLC analysis of gliadin incubated with recombinant E40 at different incubation time, up to 240 min. At time point 0, gliadin proteins were fractionated according to the hydrophobicity in ω-, α-, and γ-gliadin.

The 33-mer degradation was monitored by LC-MS/MS before (Fig. 5, panel A) and after (Fig. 5, panels B–E) E40 digestion over 0–60 minutes of incubation (0′–60′, E40:33-mer molar ratio of 1:48). Notably, a slight hydrolysis of 33-mer was observed as soon as the incubation started (Fig. 5, panel B, 0 minutes), whilst the native peptide signal completely disappeared after only 15 minutes (Fig. 5, panel C). More specifically, the MS/MS spectra analysis demonstrated that E40 activity broke down all the 33-mer immunotoxic sequences (Fig. 5, panel F). E40 cleaving site occurred between F-P and Q-L, leading to short peptides lacking of T-cell stimulatory capacity^6^. This finding was also observed at more diluted E40 concentration (1:96, molar ratio), and complete 33-mer degradation took 30 minutes (not shown). Further, the ability of E40 to hydrolyze harmful peptides in whole gliadin proteins was assessed by HPLC analysis (Fig. 6). Because of the complex mixture of whole gliadins, digestion with E40 (1:48, molar ratio) was extended up to 240 minutes of incubation (Fig. 6, panels B–F). Undigested gliadin sample was run as reference control (Fig. 6, panel A). As expected, the chromatographic profile was drastically changed, since peaks assigned to α-, β-, and γ-gliadin were markedly reduced after 30 min (panel C) and disappeared between 60 and 120 min of E40 digestion (panel D and E). The profiles of digested products at 180 min and 240 min (panel F and G) were similar, indicating that no further degradation arose.

### Assessment of E40 proteolytic degradation of humoral and T-cell gluten epitopes by G12 ELISA and celiac mucosa T cells

We next evaluated whether E40 enzymatic activity efficiently neutralizes: i) the capacity of gluten proteins to bind G12 antibodies, and ii) the stimulation of a T-cell-mediated immune response, measured by IFN-γ release. To this purpose, gliadin proteins were digested with E40 alone, or with E40 co-incubated with the gastrointestinal proteases pepsin and trypsin/chymotrypsin, at different pH and time points as detailed in Table 1. To estimate the effect of E40 digestion on the immunological potential of wheat, gluten content of samples A-L was determined by the monoclonal antibodies specific for QPQLPY sequence (Elisa – G12)^25^. Compared to untreated gliadin samples (I and L), E40 digested sample (from A to H) showed a drastic reduction of gluten content, well below 20 ppm (Table 1).

**Table 1:** Enzymatic digestion scheme of gliadin proteins by pepsin-trypsin/chymotrypsin and E40, and determination of gluten content of digested gliadin sample A-L (see Table 1) by G12-Elisa assay.

Gliadin digest sample	Gastric hydrolysis condition	Duodenal hydrolysis condition	ppm (ng/mL)
A	E40 (1 h) (pH 4)		1.72
B	E40 (2 h) (pH 4)		2.26
C	E40 + Pepsin (1 h) (pH 4)		5.23
D	E40 + Pepsin (2 h) (pH 4)		5.42
E	E40 + Pepsin (2 h) (pH 4)	Trypsin + Chymotrypsin (2 h) (pH 6)	4.09
F	E40 + Pepsin (2 h) (pH 4)	Trypsin + Chymotrypsin (4 h) (pH 6)	2.68
G	E40 + Pepsin (1 h) (pH 4)	Trypsin + Chymotrypsin (2 h) (pH 6)	1.68
H	E40 + Pepsin (1 h) (pH 4)	Trypsin + Chymotrypsin (4 h) (pH 6)	2.05
I	Pepsin (2 h) (pH 4)	Trypsin + Chymotrypsin (4 h) (pH 6)	>800
L	Pepsin (2 h) (pH 4)	Trypsin + Chymotrypsin (4 h) (pH 7)	>800

**Figure 7:**
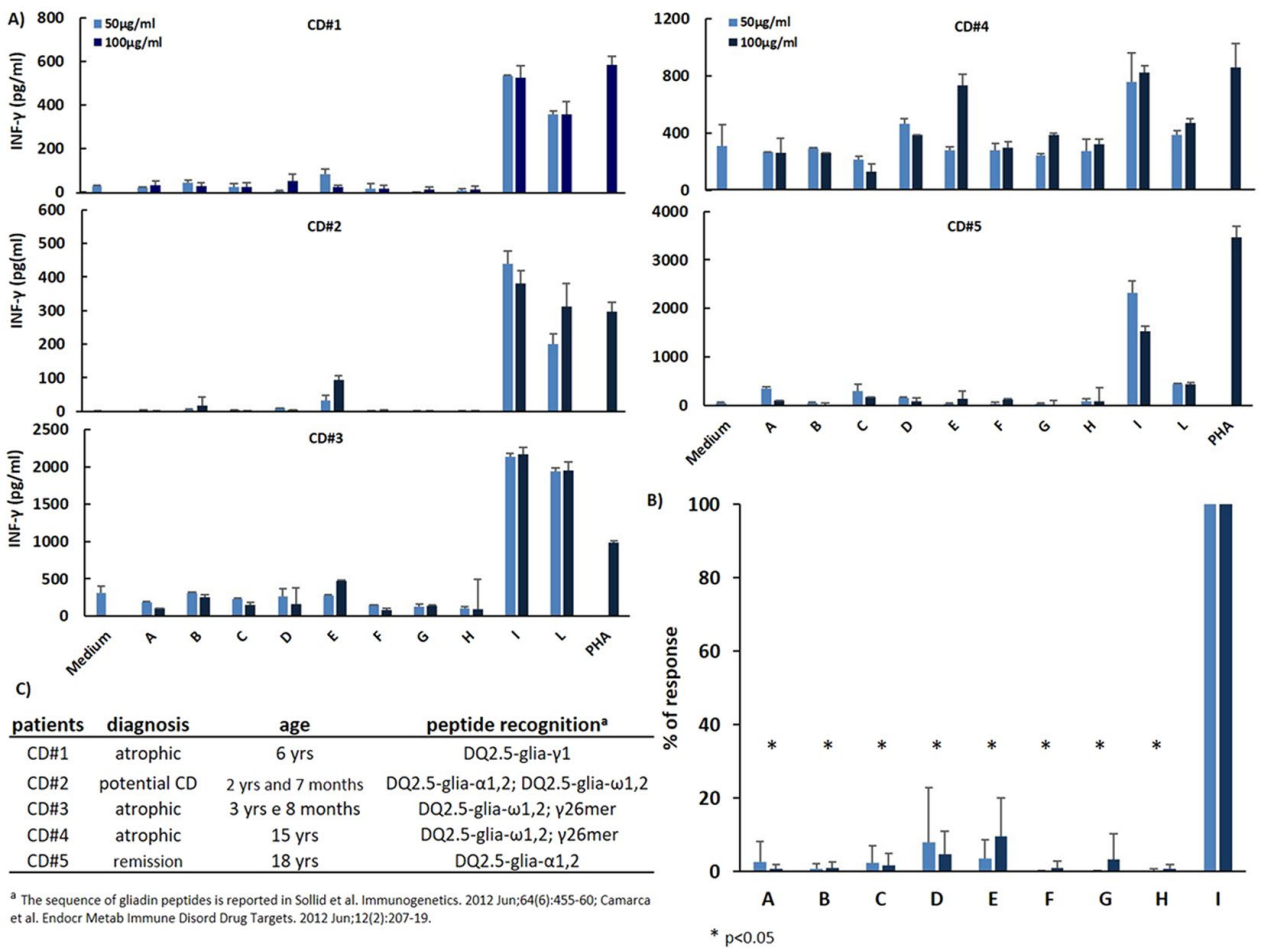
The digestion with E40 strongly reduced the immunostimolatory capacity of gliadin on intestinal T cells. Gliadin purified from hexaploid wheat was treated with E40 in the absence (samples A and B) and in presence of gastrontestinal proteases (samples C–E) at the conditions specified in Table 1. The enzymatic digests of gliadin were next deamidated by tTG treatment and assayed for the stimulatory capability on intestinal T cells. T-cell activation was determined by the measurements of IFN-γ production. All gliadin digests were assayed at 50 and 100 μg/ml. (**A**) Gliadin reactive T-cell lines obtained from jejunal biopsies of 5 different celiac subjects were assayed for recognition of E40-treated gliadin (samples A–H). Autologous EBV-transformed B cells were used as antigen presenting cells. Untreated gliadin digests (samples I and L) and phytohemagglutenin (PHA) were used as positive controls. Each panel is a representative experiment out of three done for each T-cell line. (**B**) Percent of T-cell response to E40-treated gliadin digests were calculated on the E40-untreated gliadin digests (sample I). The data are mean responses of T-cell lines from 5 different CD patients. (**C**) Characteristic of CD subjects enrolled in the study as source of gliadin-reactive intestinal T-cell lines. The profile of gliadin peptides recognized by each T-cell line is reported. Unpaired Student T test was used to assess the statistical significance. *p < 0.05.

In order to evaluate the capability of gliadin preparations to activate a T-cell response, different gliadin enzymatic digests (sample A-L) were deamidated by tTG treatment, before being assayed on T cells. Highly responsive to gliadin epitopes, CD4+ T-cell lines previously established from intestinal biopsies of CD patients, were used as sensitive bioassay to test E40 detoxifying properties. Five different T-cell lines were selected on the base of their recognition pattern of gliadin peptides, covering the most immunogenic epitopes from all three gliadin families (α-, γ- and ω-gliadins) (Fig. 7, panels A–C). As expected, all the T cells lines produced high level of IFN-γ when exposed to pepsin-chymotrypsin/trypsin digested gliadin (samples I and L). Conversely, in all the experimental conditions examined, no detectable IFN-γ production was measured in T cells exposed to E40-digested gliadins. To unequivocally exclude that inhibited IFNγ production might result from an unspecific toxic response to E40, peripheral blood mononuclear cells (PBMCs) from healthy donors were stimulated with the mitogen phytohemagglutinin (PHA), in presence or absence of E40-gliadin digests, at different experimental conditions. No substantial inhibition of IFN-γ production in PBMCs occurred because of exposure to E40-gliadin digests (Fig. S5). Therefore, neutralization of the immune-stimulatory properties of gliadin peptides on human T cell, otherwise defined as gliadin detoxification, was specific to E40 proteolytic activity.

## Discussion

It has been widely demonstrated that an abnormal immune response to gluten occurs in subjects with CD. HLA genes are the main predisposing factor for CD^26^, although the risk genes are necessary but non-sufficient to determine the disease, and several environmental factors are required^13^. In particular, gastrointestinal viral infections, factors disrupting gut barrier integrity and intestinal dysbiosis have a role in the incidence, progression and management of gluten intolerance^13^. The upstream cause is the incomplete hydrolysis of gluten proteins by human gastro-intestinal proteases. The pronounced resistance of gluten proteins to digestive process is not due to enzymatic deficiencies in celiac patients but rather to gluten’s peculiar primary sequence, enriched of proline-glutamine residues, inefficiently cleaved by gastrointestinal proteases^6^. The oral supplementation of exogenous endopeptidases, able to act synergistically with the physiological proteases in degrading gluten proteins, has been suggested as one of the most effective strategy to support gluten-free dietary treatments, in particular in case of inadvertent (unintentional) exposure to gluten. Several proteolytic enzymes are currently under investigations for their ability to degrade gluten under gastric conditions, and suitable for oral enzyme supplementation in CD. Particularly, three endoproteases have been more extensively tested for their efficacy in abolishing or reducing the T-cell immune-stimulatory capability^27–29^. A fast proteolytic activity at low pH (as occurring in the stomach), pepsin-resistance, and high specificity for breaking down proline and glutamine rich sequences characterizing the immunotoxic gluten peptides are the desired properties of optimal glutenases in OET. These combined properties should prevent that gluten peptides may reach the duodenal mucosa having maintained their immune-stimulatory form^30^. Latiglutenase (ImmunogenX, previously ALV003) is a combination of the glutamine-specific endoprotease EP-B2 degrading complex gluten bread proteins, and a prolyl-endopeptidase (PEP) from *Sphingomonas capsulata*, that detoxifies the residual oligopeptide products of EP-B2^31^. In a phase II trial, Latiglutenase has been shown to successfully degrade immunogenic gluten fragments in the stomach, and to attenuate mucosal injury in CD patients consuming up to 2 g gluten daily along with GFD^32^. The synthetic enzyme KumaMax30 had similar *in vitro* results to Latiglutenase, but is still under development^29,33^. AN-PEP, an *Aspergillus niger* derived prolyl-endopeptidase, had very promising *in vitro*, *ex vivo*, and *in vivo* results, and its safety has been recently demonstrated in clinics^28,34^.

In this promising scenario, we report the identification of the novel microbial Endopeptidase 40, that shows to be endowed with all the glutenasic properties needed for successful OET in CD. E40 was recombinantly expressed in the actinomycete heterologous host *S*. *lividans* TK24, a strain free from endogenous plasmids and lacking an outer membrane, therefore devoid of lypopolysaccharide endotoxins^35^.

The recombinant expression of E40 in *S*. *lividans* TK24 resulted in the secretion of active E40, confirming that the wild signal peptide has been recognized and the enzyme has been processed to maturation, exactly as done by the wild producer *Actinoallomurus* A8. As expected, all the chemico-physical characteristics and biological properties of the native form were maintained, e.g. stability and activity at post-prandial gastric pHs, resistance to pepsin digestion, high efficiency in the extensive degradation of the most immunogenic peptide of α-gliadin, such as the Pro-Gln enriched 33mer peptide (LQLQPFPQPQLPYPQPQLPYPQPQLPYPQPQPF), as well as of whole gliadin proteins^4,30^. MS/MS spectra analysis demonstrated E40 to break down all the six 33-mer immunotoxic epitopes, displaying an extremely fast cleaving activity at covalent bond on F-P and Q-L residues within 15 minutes of treatment, with no 33-mer signal detectable within 1 hour. Also whole gliadin proteins from hexaploid wheat were markedly proteolized within 1 hour of E40 digestion. The absence of immunogenic potential of the pool of peptidic fragments produced during E40 digestion of wheat gliadin was assessed via G12 moAb competitive ELISA, specific to detect gluten immune-stimulatory peptides having the QPQLPY sequence. The validated human T-cell assay further confirmed that no residual immune-toxicity remains in E40–digested gluten.

Noteworthy, E40 gluten-degrading activity is independent from pepsin proteolysis. Our findings demonstrated that E40 conveys the desired property to get a fast gluten gastric digestion, thus speeding up the breaking down of immunotoxic peptides particularly into the stomach. This observation, and the comprehensive microbiological, proteomic and immunological analyses performed in our study, candidates E40 to be a fast-acting and strongly efficient glutenase. Due to the fact that the GFD is indeed restrictive for many celiac subjects that are not compliant with the dietary therapy, the availability of E40 as an adjuvant enzymatic compound favoring the complete and rapid digestion of gluten proteins would be of great benefit to people in need of adhering to a GFD, but anyhow exposed to the risk of inadvertent gluten exposure. Further *in vivo* and human studies are currently planned to confirm the efficacy of E40-based OET for CD. Furthermore, it will be of interest to also assess E40 utility in the dietary management of other gluten-related disorders^36^. In this context, it is noteworthy that *Streptomycetes*, used to produce the recombinant E40, are regarded as a safe source of proteins for human alimentary use. Two examples of food enzymes sourced from *Streptomyces* spp are: glucose isomerases used for fructose syrup production^37^, and the widely exploited transglutaminase from *S*. *mobaraensis*, used in food industry for its properties in improving the texture and overall quality of final food products, such as processed meat and fish products, as well as diary and baked food^38^.

In conclusion, we have provided evidences the new E40 glutenase efficiently degrades the most immune-toxic gluten peptides, at acidic and gastrointestinal simulating conditions. The availability of adjuvant enzymes capable of effectively degrading gluten proteins during their transit into the first gastrointestinal tract, and particularly into the stomach, would have a great relevance to prevent the adverse inflammatory response induced by gluten, in intolerant individuals^4^.

## Materials and Methods

### Reagents

Actinomycete strain *Actinoallomurus A8* was from the collection of “Fondazione Istituto Insubrico di Ricerca per la Vita” laboratory. The N-Succinyl-Ala-Ala-Pro-Phe p-nitroanilide (suc-Ala-Ala-Pro-Phe-pNA) and N-Succinyl-Ala-Ala-Pro-Phe-7-aminomethyl coumarine (suc-Ala-Ala-Pro-Phe-AMC) were from Bachem (Bubendorf, Switzerland), pepsin, trypsin, nalidixic acid, and apramycin were from Sigma-Aldrich (Milan, Italy), mannitol was from Carlo Erba (Cornaredo, Italy), soya flour was from Cargill (Padova, Italy), agar and soy peptone were from Conda (Madrid, Spain), sucrose and glucose were from VWR (Leuven, Belgium), yeast and malt extracts were from BD (Franklin Lakes, NJ, USA) and Costantino (Favria, TO, Italy) respectively, the 33-mer a-gliadin peptide was synthesized by Biotem (Apprieu, France). All other reagents used for cloning of the endoprotease were of analytical reagent grade. Strain *S*. *lividans* TK 24 and expression vector pIJ86 were from the John Innes Center (Norwich, UK). Protein concentration was measured by BCA assay (Thermo Scientific, Rodano, MI, Italy).

### Enzyme activity assay

Enzymatic activity was performed in 96-well microtiter plates (transparent, flat-bottom) using the Infinite 200 PRO plate reader (Tecan, Männedorf, CH). Twenty microliters of enzyme samples (concentration range 10–50 nM) were pre-incubated for 5 min at 37 °C and then added to 180 µl of 220 µM suc-Ala-Ala-Pro-Phe-pNA in citrate phosphate buffer (0.1 M citric acid, 0.2 M disodium hydrogen phosphate, pH 5). Samples were incubated for 20 minutes at 37 °C. The pNA was detected at 410 nm, reading at interval of 5 min. One Unit was defined as the amount of enzyme that released 1 µmol of pNA per minute, sample activity was expressed as enzyme Units per ml of supernatant or per mg of protein.

The pH optimum was determined using the same substrate which was prepared in 0.2 M ammonium citrate for pH 2 or citrate phosphate buffer at pH range 3–8, respectively.

### Wild Endopeptidase E40 discovery and gene identification

Wild E40 was produced as extracellular protein by submerged fermentation of strain A8, an actinomycete strain belonging to the genus *Actinoallomurus*, well growing at pH close to 5^21^. The discovery and purification of Endopeptidase E40, as well as the cloning/expression and purification of its recombinant form are described in Supplementary Material.

### Zymography analysis

E40 containing samples were run by non-reducing SDS-PAGE (12% polyacrylamide) at 100 V using a Tetra-cell Mini-PROTEAN systems (Bio-Rad, Milan, Italy). Gel was washed twice with citrate-phosphate/phosphate buffer (pH 5.0), and then incubated with the same buffer including 100 µM of suc-Ala-Ala-Pro-Phe-AMC. The activity of E40 was visualized in the gel exposed by UV-light.

The proteolytic activity of E40 was also evaluated by using gelatin as substrate: after electrophoretic running, PA-gel was washed with citrate phosphate buffer pH 5.0 and overlapped onto a zymogram 10% gelatin gel (Bio-rad) equilibrated with the same buffer for 10 minutes. The two overlapped gels were incubated at 37 °C for 2 hours, then the gelatin gel was stained with Coomassie Brilliant R250. Gelatin digestion was visualized after gel destaining as clear lysis bands, due to proteolytic action of proteases diffused from the PA-gel to the zymogram one.

### E40 stability to digestive (pepsin/trypsin) proteases

The enzymatic activity of purified E40 was evaluated in the presence of pepsin or trypsin. Aqueous solutions of enzymes, alone or in combination, (0.2 mg/ml E40, 0.1 mg/ml Pepsin or Trypsin) were diluted in citrate-phosphate buffer containing suc-Ala-Ala-Pro-Phe-pNA at pH 4, 4.5 and 5 for pepsin, and pH 6, 7, and for trypsin assay (1–10 nM E40 final concentration). Reactions (200 µl volume in 96-wells flat bottom µtiter plates) were protracted for 110 minutes at 37 °C. Absorbance was monitored every 10 min for determining the enzymatic activity. Pepsin and trypsin without E40 were processed in the same way and tested in the same condition as reference control. Each analysis was carried out in duplicate.

### Digestion of 33-mer by E40

Digestion was performed in U-bottom 96-wells microtiter plate. 33-mer was diluted in citrate phosphate buffer (pH 5) to 5 mg/ml, mixed with E40 diluted in the same buffer (1:48 enzyme vs substrate molar ratio) and incubated at 37 °C. At the time point 0, 15, 30 and 60 minute aliquots (10 µl) were taken, and reactions were stopped by boiling for 3 minutes before being analyzed by LC-MS/MS.

Samples were analyzed by HPLC system Accela Instrument (Thermo Fisher Scientific, San Jose, CA) coupled to both UV detector and LTQ-XL ion trap mass spectrometer (Thermo Fisher Scientific). Samples were diluted with 90 μl Milli-Q water and 10 μl loaded through a column Aeris Peptide 3.6 μm XB-C18 150 × 2.1 mm, (Phenomenex, Torrance CA, USA). Eluent A was 0.1% acetic acid (v/v) in Milli-Q water, eluent B was acetonitrile. The separation was carried out at a flow rate of 0.2 mL/min (room temperature), with a linear gradient from 10% to 40% of solution B over 15 minutes. The column effluent was split to give a flow rate of 80 μl/min and 20 μl/min into the UV detector and ESI source respectively. The mass spectrometer operated in data-dependent mode and all spectra were acquired in the positive ionization mode with an m/z scan range of 200e2000. MS and MS/MS spectra were elaborated using the Proteome Discoverer 2.0 (Thermo Fisher).

### Hydrolysis of gliadins by E40

Gliadins were extracted from whole flour of *Triticum aestivum* (Sagittario cultivar) according to Gianfrani *et al*.^8^. Gliadin (1 mg) were dissolved in 1 ml of 0.1 M ammonium acetate pH 4 and incubated with E40 enzymes (enzyme:substrate, 1:20), at 37 °C for different time points (0, 15, 30, 60, 120, 240 minutes). The enzymatic hydrolysis was stopped by boiling for 5 minutes. Sample were lyophilized and stored at −40 °C until further chemical analysis.

SDS-PAGE was performed on a Tetra-cell Mini-PROTEAN systems (Bio-Rad, Milan, Italy). Digested gliadin samples were dissolved in Laemli buffer (0.125 M Tris–HCl pH 6.8, 5% SDS, 20% glycerol, 0.02% bromophenol blue) and loaded onto precast 12% acrylamide gel (Bio-Rad). Electrophoresis was carried out under non-reducing conditions, omitting β-mercaptoethanol in the Laemli buffer. Protein bands were visualized with silver blue (Coomassie Brilliant Blue G-250) and digitalized using a LABScan scanner (Amersham Bioscience/GE Healthcare, Uppsala, Sweden).

RP-HPLC was carried out on a RP-HPLC using an HP 1100 Agilent Technology modular system (Palo Alto, CA, USA). Digested gliadin samples were suspended in 0.1% TFA and separated by C18 column (Aeris PEPTIDE, 3.6 μm, 250 × 2.10 mm i.d., Phenomenex, Torrance, CA, USA). Eluent A was 0.1% TFA (v/v) in Milli-Q water, eluent B was 0.1% TFA (v/v) in acetonitrile. The column was equilibrated at 5% B. Peptides were separated applying a linear 5–70% gradient of B over 90 minutes at a 0.2 mL/min flow rate. Chromatographic separation was performed at 37 °C, using a thermostatic column holder. The column effluent was monitored at 220 and 280 nm with an UV-Vis detector.

### Hydrolysis of gliadin proteins by pepsin-trypsin/chymotrypsin and E40 and deamidation by tTG

Gliadin were suspended in 1 ml of 0.1 M ammonium acetate and digested as the scheme shown in Table 1. All enzymes were added in w/w ratio 1:20 (enzyme:protein) and incubated at 37 °C at indicated pH and incubation time. E40/pepsin/chymotrypsin-digested gliadins were deamidated by tTGase (Sigma Aldrich), as previously described^8^. Briefly, the enzymatic gliadin digests (500 μg/ml) were incubated with 2 U of guinea pig tTG (T-5398, Sigma, St. Louis, MO, USA) at 37 °C for 2 hours in PBS with 1 mmol/L CaCl2.

### T-cell immunogenicity assays

Gliadin-reactive T-cell lines (TCLs) were previously generated from duodenal biopsies of HLA-DQ2 CD patients. Briefly, intestinal mononuclear cells were stimulated with irradiated autologous peripheral blood mononuclear cells (PBMCs) and deamidated pepsin-chymotrypsin digested gliadin extracted from the hexaploid wheat. Growing T cells were kept in culture by repeated stimulation with heterologous irradiated PBMCs and PHA, and IL-2 as a growth factor. The peptide specificity of the TCLs was evaluated by assaying their reactivity toward a panel of immunogenic gluten peptides, and revealed a large repertoire of peptide recognition^39,40^. In the functional assays, the immune response of TCLs to gliadin enzymatic digests (samples A-L, Table 1) was assayed by detecting IFN-γ production. T cells (3 × 10^4^) were co-incubated with irradiated autologous EBV-transformed, B-lymphoblastoid cell lines (B-LCLs, 1 × 10^5^), in 200 μL of complete medium (*X*-*Vivo*15 supplemented with 5% human serum and penicillin-streptomycin antibiotics, all reagents supplied by Lonza-BioWhittaker, Verviers, Belgium) in U-bottom 96-well plates. After a 48 hours incubation, cell supernatants (50 μL) were collected for IFN-γ determination. Each antigen preparation was assayed in duplicates and in at least three independent experiments for each T-cell line. IFN-γ was measured by a classic sandwich ELISA using purified and biotin-conjugated anti-IFN-γ Abs purchased from Mabtech (Nacka Strand, Sweden). The sensitivity of the assay was 32 pg mL^−1^. This study with celiac patients derived TCLs was approved by the ethical committee of SG. Moscati Hospital, Avellino Italy (Register CECN/819 dated 03/21/2018), and Department of Pediatrics University Federico II of Naples (Register 343/17 dated 01/30/2018). All experiments were performed in accordance with relevant ethical guidelines and regulations. Informed consent for study participation was obtained for all enrolled patients.

### Evaluation of gluten by G12 competitive ELISA

Gluten content (samples A-L, Table 1) was measured by G12 competitive ELISA, using a Ridascreen® Gliadin Elisa Kit (R-Biopharm AG Darmstadt, Germany) according to the manufacturer’s and to the AOAC guidelines.

## Supplementary information

supplementary data
