# Supplementary material for: E40, a novel microbial protease efficiently detoxifying gluten proteins, for the dietary management of gluten intolerance
Source: Sci Rep. 2019 Sep 11;9:13147. doi: 10.1038/s41598-019-48299-7 (PMC6739405; doi:10.1038/s41598-019-48299-7)
Supplement: Supplementary file 1 — supplementary data [file 41598_2019_48299_MOESM1_ESM.docx]

**Supplementary Information of**

**E40, a novel microbial protease efficiently detoxifying gluten proteins, for the dietary management of gluten intolerance.**

Linda Cavaletti,^1^ Anna Taravella,^1^ Lucia Carrano,^1^ Giacomo Carenzi,^1^ Alessandro Sigurtà,^1^ Nicola Solinas,^1^ Salvatore De Caro,^2^ Luigia Di Stasio,^2^ Stefania Picascia,^3^ Mariavittoria Laezza,^3^ Riccardo Troncone,^4^ Carmen Gianfrani,^3,4*^ Gianfranco Mamone.^2^

^1^ Fondazione Istituto Insubrico di Ricerca per la Vita (FIIRV), Gerenzano, Varese, Italy.

^2^ Institute of Food Sciences, National Research Council, Avellino, Italy.

^3^ Institute of Biochemistry and Cell Biology, National Research Council, Naples, Italy.

^4^ Department of Translational Medical Science (Section of Paediatrics), and European Laboratory for the Investigation of Food-Induced Diseases, University “Federico II”, Naples, Italy.

^*^ Corresponding Author: [c.gianfrani@ibp.cnr.it](mailto:c.gianfrani@ibp.cnr.it)

**Endopeptidase E40 discovery.** A screening process planned to discover new wild microbial enzymes suitable as CD OET candidates was carried out. Newly isolated acidophylic actinomycetes, stored in the Fondazione Istituto Insubrico di Ricerca per la Vita strain collection, have been chosen as target source for this project, and *in-vitro* activity assays coherent with the OET needs have been used to identify potential novel glutenases present in strain cultures supernatants. Briefly, extracellular proteases active at acidic pH were highlighted by in-vitro assays, using simple commercial chromo/fluorogenic substrates; positive samples were then tested for ability to degrade 33mer in the same conditions. Once identified promising samples, corresponding producing strains were re-fermented for confirmation of extracellular proteases production, activity driven crude purification of the protein and assessment of it potential glutenase activity. Through this process, strain A8, belonging to the genus *Actinoallomurus,* was selected for the investigation that allowed the discovery of E40. A schematic representation of the whole discovery process is given in Figure S1.

**Strain A8 cultivation and wild E40 identification.** Strain A8 was maintained on ISP3 agar medium (Shirling and Gottlieb, 1966) acidified at pH 5,5 with HCl (Figure S2). The microbial content of one plate was scraped and inoculated into one 50 ml Erlenmeyer flask containing 15 ml of medium AF5 (g/l: dextrose 20, yeast extract 2, soybean meal 8, NaCl 1 and 2-(N-morpholino)-ethanesulfonic acid 10; pH adjusted to 5,5). The inoculated flask was grown at 28°C, on a rotary shaker operating at 200 rpm. After 5 days of incubation, the culture was inoculated into a second series of 500 ml Erlenmeyer flasks containing 100 ml of the same fermentation medium and incubated for several days in the same conditions. Proteolytic activity in the supernatant culture was monitored during the course of fermentation; released AMC was measured with a Fusion micro-plate reader (Perkin Elmer) at 360 nm (excitation) and 460 nm (emission) wavelength.

The fermentation was harvested after 15 days and the broth culture was centrifuged at 4000 rpm for 15 minutes. The culture supernatant was filtered off to remove residual microbial bodies, and 200 ml of the filtrate was further centrifuged at 10000 rpm for 20 minutes at 4°C to remove the insoluble fraction.

Proteins were precipitated from supernatant by ethanol (1:4 v/v); after removing of the soluble fraction, pellet was air-dried and proteins were resuspended in 0.2M Ammonium Citrate buffer pH 5, and checked for activity toward the substrate suc-Ala-Ala-Pro-Phe-AMC. Resuspended proteins were chromatographed on ionic exchange resin Amberlite IRA900 (Alfa Aesar GmbH, Karlsruhe, 76185 Germany); the obtained fractions were tested for activity and the active ones were pooled. Proteins were further fractionated by size exclusion filtration on different molecular weight cut-off (Vivaspin Sartorius GmbH 37070 Goettingen, Germany). The 100 kDa ultrafiltrate sample, fraction “<100 kDa”, showed to be active. and corresponding to the new endoprotease; it was used for glutenasic activity characterization. One aliquot of the same sample was boiled and submitted to MudPit analysis (CNR service, Milan, Italy). Identified peptides where compared to public protein databases (no significant matching results) and to proteins inferred by *Actinoallomurus* A8 genome translation (CNR service, Milan, Italy) which allowed identification of the E40 coding gene (e40), submitted to GenBank (<https://www.ncbi.nlm.nih.gov>) with accession number MK303398 (Figure S3).

**Cloning and expression of E40 coding gene.** The e40 open reading frame was amplified from *Actinoallomurus* A8 genomic DNA using Phusion High Fidelity polymerase (Thermo Scientific, Rodano, MI, Italy) and 0.5 μM of E40 his-tag Fw (5′- AAA**AAGCTT**CAGTGGTGGTGGTGGTGGTGTCCGAAGGCTCCGGTGCC-3′) and E40Rev (5′-AAA**TGATCA**ATGTCACGACGCGTGACCG-3′) oligonucleotide primers that introduced HindIII and BclI restriction sites (double underlined), respectively and one glycine followed by six histidine residues at the C-terminus of the protein (underlined) into the PCR product. Cycling conditions were: 3 minutes at 98°C, followed by 10 cycles of 10 s at 98°C, 20 s at 67°C and 50 s at 72°C, 20 cycles of 10 s at 98°C, 70 s at 72°C, with a final extension at 72°C for 5 minutes. The PCR products were purified from an agarose gel, digested with HindIII and BclI and ligated with pIJ86 digested with HindIII and BamHI to produce pIJ86/e40 (Figure S4). pIJ86/e40, with e40 transcribed from the strong constitutive ermE* promoter, was used to transform *E. coli* ET12567/pUZ8002. The absence of PCR-generated mutations in the plasmid thus obtained was assessed by DNA sequencing. Conjugations were performed according to Flett *et al* using *E. coli* ET12567 as the donor and *S. lividans* TK24 as the recipient. *^1^* The mating mixture was spread onto Mannitol Soya flour (mannitol 8 g/L, soya flour 8 g/L, agar 8 g/L, MS) agar plates containing 10 mM MgCl_2_ and incubated at 28 °C. After 16-20 h, 1ml distilled water containing 50 μg/ml nalidixic acid and 30 μg/ml apramycin was added to the surface of each plate and spread using a glass rod and further incubated at 28 °C until exconjugant colonies appeared. The exconjugants were repeatedly plated on to MS agar containing nalidixic acid and apramycin, a final spore suspension, prepared according to Kieser *et al,* was stored at -80°C in 20% glycerol. ^2^

**Recombinant production of E40 by *S. lividans* TK24 submerged fermentation at 100 ml or 15L scale.** *S. lividans* TK24/plJ86/e40 spore suspension was inoculated in Erlenmeyer flasks (50 ml) containing 20 ml of Medium V (glucose 20 g/L, yeast extract 5 g/L, soy peptone 10 g/L, NaCl 1 g/L. pH~6.7) added with Apramycin 50 mg/L and incubated on a rotatory shaker at 200 rpm at 30°C. After 5 days of growth, the culture was inoculated in one Erlenmeyer flask (500 ml) containing 100 ml of the Medium P (Sucrose 340 g/L, Glucose 20 g/L, Yeast extract 3 g/L, Soy peptone 5 g/L, Malt extract 3 g/L, pH 6.7) added with Apramycin 50 mg/L and incubated in the same conditions for 8 days. For 15 L scale fermentation, the 5-day culture grown in Medium V was inoculated in three 500 ml-Erlenmeyer flasks containing 100 ml of the Medium V, and incubated in the same conditions. After 72h, the flask cultures were harvested, pooled and used to inoculate a fermenter (Biostat Cplus, Sartorius Stedim, Goettingen, Germany) containing 15 liters of Medium P added with Apramycin 50 mg/L. Fermentation was run at 30°C under stirring conditions of 450 rpm, E40 production was monitored over the time by measuring the enzymatic activity in the culture supernatant after centrifugation at 11000g for 6 min. Fermentation was stopped after 94 hours, when enzymatic activity reached 1430 U per ml of supernatant. The harvested *S. lividans* TK24 plJ86/e40 culture was centrifuged for 90 min at 4120g and the supernatant was collected and filtered on Rapida A paper (Enrico Bruno, Turin, Italy) and further clarified two times onto polyethersulfone Opticap® capsules (nominal pore sizes 1.0 µm and 0.5 µm) (Merck, Vimodrone, Italy). Next, the clarified solution was concentrated 10 times by ultrafiltration system (TFF1) using Pellicon 3 Ultracel TFF cellulose cassettes (10 kD nominal MW cutoff) and added to 0.5 M NaCl and 50 mM Na_2_HPO_4_ pH 7.2. Enzyme was purified by Immobilized Metal Affinity Chromatography (IMAC) by using Ni Sepharose® 6 Fast Flow resin (GE Healthcare, Milan, Italy). IMAC column was equilibrated with 5 volumes of phosphate buffer (pH 8.0), elution was carried out by 5 volumes of 250 mM imidazole, 50 mM phosphate buffer (pH 8.0), eluted fractions (330 ml) were adjusted to pH 6.3 with formic acid and depigmented by DEAE anion exchange chromatography (GE Healthcare). Depigmented samples were concentrated and desalted by ultrafiltration system (TFF2, Pellicon, 10 kD MW nominal cut-off). Before freeze-drying, sample was added to mannitol and trehalose (15 and 5 mg/m respectively), to improve the crystallization process.^3^ The protein content of purified sample was 6% (measured by BCA assay) with activity of 41000 U/mg protein. Sample was stored at -20° C until further use.

**Cytotoxicity assay**. Peripheral blood mononuclear cells (PBMCs) were isolated from blood samples of healthy donors by Ficoll-Paque (GE Healthcare, UK) density centrifugation, according to the manufacturer’s instructions. PBMCs were seeded (2x105) in a 96-well plates in complete medium and incubated at 37°C and 5% CO2 with digested gliadin samples (50 µg/ml, samples A-L, (Table 1, Figure 7) with or without phytohemagglutinin (PHA, 2 µg/ml). Cell supernatants (50 µl) were collected after 48 hours for evaluation of IFN-γ by ELISA assay. Samples were assayed in duplicates (Figure S5).

**References:**

1. Flett, F., Mersinias, V., Smith, C. P. High efficiency intergeneric conjugal transfer of plasmid DNA from Escherichia coli to methyl DNA-restricting streptomycetes. *FEMS Microbiol. Lett.* **155**, 223–229 (1997).
2. Kieser, T., Bibb, M.J., Buttner, M. J., Chater, K. F., Hopwood, D. A. *Practical Streptomyces in Genetics*. (John Innes Foundation, 2000).
3. Jena, S., Suryanarayanan, R., Aksan, A.l Mutual Influence of Mannitol and Trehalose on Crystallization Behavior in Frozen Solutions. *Pharm Res.* **33**, 1413-25 (2016).


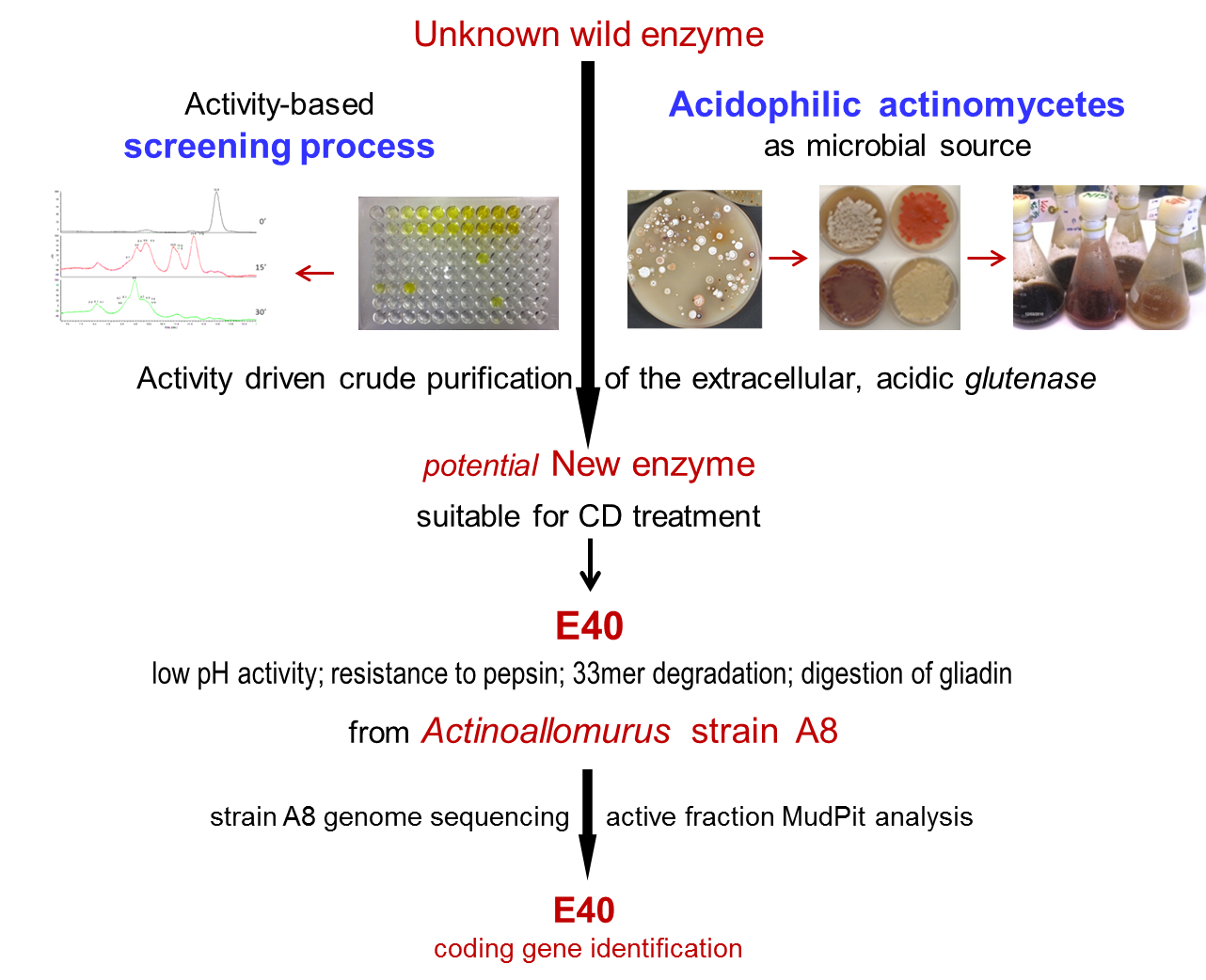


**Figure S1**. Discovery process of Endopepeptidase E40 from acidophilic actinomycetes


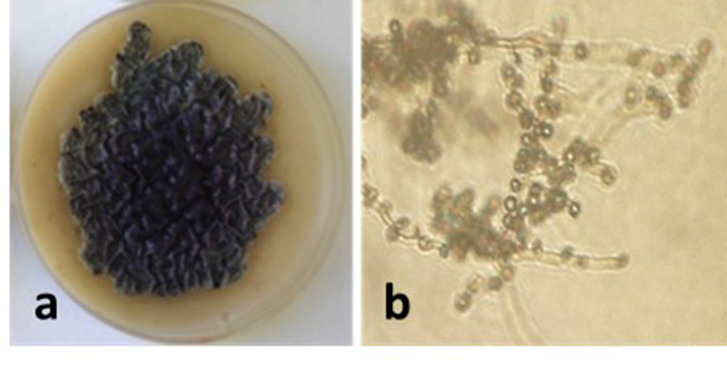


**Figure S2.** a) appearance of strain *Actinoallomurus* A8 grown on acidic ISP3 medium (Shirling and Gottlieb, 1966). b) aerial morphology on HSA5.5 medium (Humic acid Salts Agar, modified from ref. Suzuki et al., 2001) showing aerial hyphae bearing spore chains resembling sporangia (400x magnification).


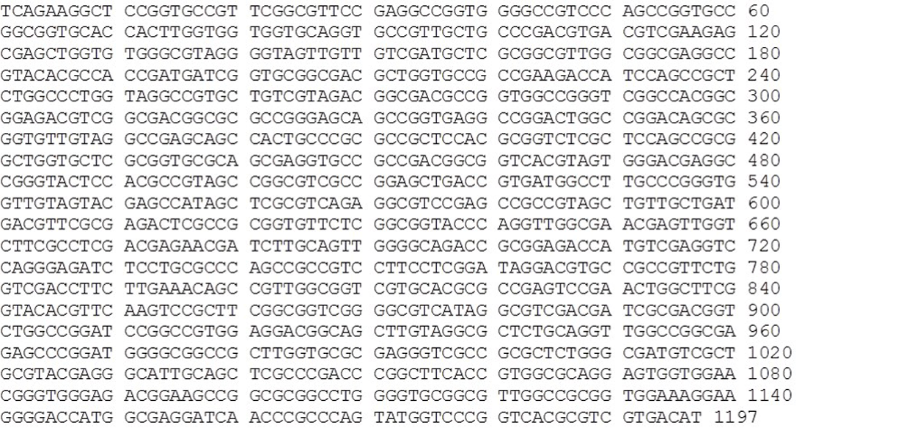


**Figure S3**. Endopepeptidase E40 whole coding gene sequence (GenBank MK303398)


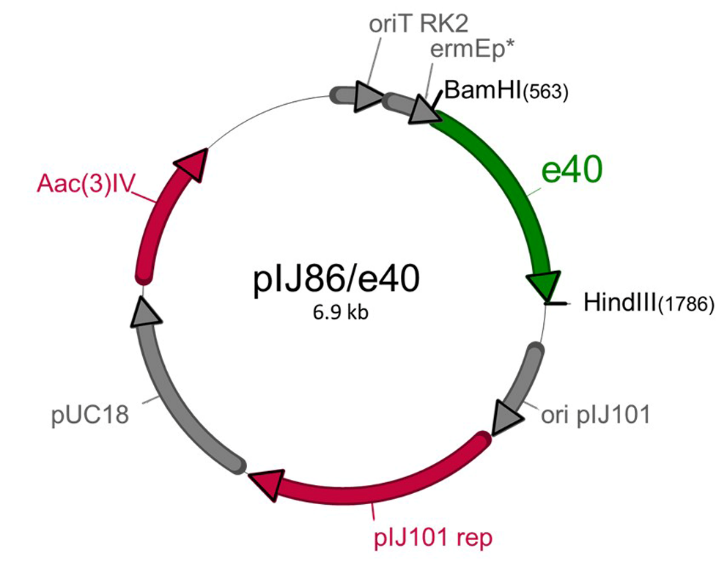


**Figure S4**. Map of plasmid pIJ86/e40


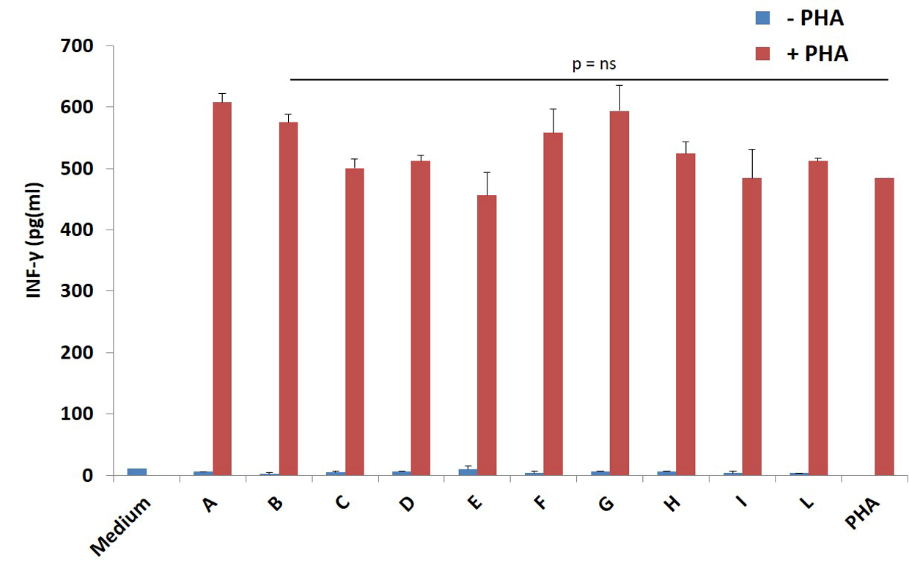


**Figure S5**. **The digestion of gliadin with E40 has no cell toxicity.**

Purified gliadin was digested with gastrointestinal proteases in the presence or absence of E40, as indicated in Table 1. Gliadin digest (samples A-L) were deamidated by tTG treatment and tested for cytotoxicity on peripheral blood mononuclear cells (PBMCs) from healthy donors. PBMCs were stimulated with gliadin samples at the concentration of 100 μg/ml in the presence or absence of the mitogen phytohemagglutenin (PHA). After 48 hours of incubation, the T-cell activation was evaluated by the detection of IFN-γ released by ELISA. In the figure is shown a representative experiment out of three performed.
